# 2263: The effect of family history, alcohol expectancies, and sex differences on hangover symptoms following intravenous alcohol self-administration in nondependent drinkers

**DOI:** 10.1017/cts.2017.123

**Published:** 2018-05-10

**Authors:** Corbin Daniel Ester, Bethany Stangl, Aruna Gogineni, Lauren Blau, Vatsalya Vatsalya, Vijay Ramchandani

**Affiliations:** National Institutes of Health, New York, NY, USA

## Abstract

OBJECTIVES/SPECIFIC AIMS: The current study examined hangover following IV alcohol self-administration (IV-ASA) using the Computer-Assisted Infusion System. The goal of the study was to identify predictors of hangover, including drinking history, alcohol sensitivity, family history, expectancies, and sex differences in nondependent drinkers. METHODS/STUDY POPULATION: The study sample included 89 healthy, nondependent drinkers aged 21–45 years. After a screening to exclude any medical illness or psychiatric disorders, participants completed an IV-ASA session. Each session consisted of a 25-minute priming phase, during which participants were prompted to press a button to receive individually standardized alcohol infusions, followed by a 2-hour “open bar” phase, during which they were instructed to recreate a typical drinking experience. Results from the IV-ASA included peak and average BrAC. Drinking patterns were assessed using the Alcohol Use Disorders Identification Test, which provided 3 subscales: consumption (AUDIT-C), dependence (AUDIT-D), and harmful drinking (AUDIT-H). Subjective response to alcohol was measured using the Drug Effects Questionnaire (DEQ). The Alcohol Hangover Scale (AHS) was used to assess hangover for the period between participants’ departure from the study unit and 10 am the next morning. The Alcohol Effects Questionnaire (AEFQ) is a measure which includes 40 true/false statements about how alcohol typically makes respondents feel, and was used to measure alcohol expectancies. RESULTS/ANTICIPATED RESULTS: Results showed that 78% of participants endorsed having at least 1 hangover symptom following IV-ASA. The most commonly reported items were tired, thirsty, headache, and hangover. There was no association between hangover scores and the AUDIT-C or IV-ASA. Because alcohol consumption was not related to hangover symptoms, risky drinking behavior was examined. Results indicated that participants endorsing 4 or more items on the AUDIT-D plus AUDIT-H subscales showed significantly higher average hangover scores. Linear regression analyses indicated that alcohol hangover scores were associated with DEQ items feel, high, and intoxicated. Ongoing analyses are examining additional predictors of hangover including family history, alcohol expectancies, sex differences, and other alcohol sensitivity measures. DISCUSSION/SIGNIFICANCE OF IMPACT: The results indicated that risky drinking patterns and alcohol response measures were positively associated with hangover symptoms in non-dependent drinks, while no correlation between consumption and hangover symptoms were found. Since previous research has shown than greater subjective response is associated with heavy drinking and predictive of alcohol use disorder, it is possible that hangover symptoms is a marker of this relationship. Since the role of hangover in the transition from heavy drinking to disorder still remains unclear, it will be important to characterize this relationship between alcohol sensitivity and hangover as a function of drinking patterns. This understanding may help to prevent this transition from at-risk drinking to alcohol dependent drinking.

